# Association between Virulence Factors and Extended Spectrum Beta-Lactamase Producing* Klebsiella pneumoniae* Compared to Nonproducing Isolates

**DOI:** 10.1155/2017/7279830

**Published:** 2017-06-08

**Authors:** Mustafa Muhammad Gharrah, Areej Mostafa El-Mahdy, Rasha Fathy Barwa

**Affiliations:** ^1^Microbiology & Immunology Department, Faculty of Pharmacy, Mansoura University, Mansoura 35516, Egypt; ^2^Department of Pharmaceutical Sciences, College of Pharmacy, Princess Norah Bint Abdulrahman University, Riyadh 11671, Saudi Arabia

## Abstract

*Klebsiella pneumoniae *is considered an important opportunistic multidrug-resistant pathogen. Extended spectrum *β*-lactamases (ESBLs) and expression of a multitude of virulence factors may work in a harmony resulting in treatment failure. This study was undertaken to compare the virulence characteristics and genetic relatedness between ESBL and non-ESBL producing* K. pneumoniae. Methods*. Antibiotic sensitivity test of all isolates was determined by disc diffusion assay. Phenotypic and genotypic detection of ESBL were done. Various virulence factors and some virulence factor-associated genes were screened. Random amplified polymorphic DNA (RAPD) was employed to investigate the genetic fingerprints of ESBL from non-ESBL producing* K. pneumoniae*.* Results*. 50% of isolates were ESBL producers. A significant association was observed between ESBL production and biofilm (strong and moderate), serum resistance, and* iss* gene. Moreover, significant association between non-ESBL producers and hypermucoviscosity was identified. Dendogram analysis of RAPD profile classified* K. pneumoniae* isolates into four clusters (a, b, c, and d). Seventy-six percent of ESBL producers belonged to cluster a. In conclusion, this study suggests a correlation between ESBL production and some virulence factors. Therefore, success of treatment depends mainly on increased clinicians awareness and enhanced testing by laboratories to reduce the spread of these isolates.

## 1. Introduction

*Klebsiella pneumoniae* is responsible for many community-onset and nosocomial infections. The increasingly high level of antimicrobial drug resistance prevalence is an exaggerated problem, especially for healthcare providers.* K. pneumoniae* can confer resistance to the majority of antibiotics by applying vast amounts of resistance mechanisms, leading to high mortality and morbidity rates. Such resistant bacteria urge the importance of focusing on antimicrobial resistance. The dominant antibiotics used for treating infections today are the *β*-lactam antibiotics, which inhibit transpeptidases participating in bacterial cell wall synthesis. Unfortunately these beta-lactam antibiotics can be deactivated by *β*-lactamase enzymes [1].

Extended spectrum *β*-lactamases (ESBLs) producing bacteria are clinically and epidemiologically important, being resistant to the effects of *β*-lactam antibiotics, but are still sensitive to clavulanic acid [1]. ESBLs are now found in all Enterobacteriaceae species around the world [2]. The majority of ESBL enzymes in* K. pneumoniae* are derived from the two classical enzyme types TEM and SHV encoded by the plasmid [3]. Moreover,* Klebsiella pneumoniae* strains producing CTX-M type have increased [4].

Good analysis of sensitivity tests and proper prescription of antibiotics require screening and identification of isolates producing ESBLs [5].* K. pneumoniae* can express high level of resistance to third-generation cephalosporins by means of gaining the plasmids which harbor genes encoding ESBLs. About 20% of* K. pneumoniae* infection in intensive care units in the United States involves strains resistant to third-generation cephalosporins [6]. The fast growing resistance expressed by ESBL producers to various antibiotic families is a serious problem that narrows the therapeutic chance against ESBL producers [7].

Virulence factors (VFs) comprise mechanisms allowing pathogenic bacteria to cause infections. Genomics becomes a good tool for defining virulence factors as it can be used to recognize genes harboring specific virulence factors. However, the organism can be avirulent if only a single factor presented; sometimes the presence of various factors at the same time is required to decide the bacterial ability of causing infections [8]. Many virulence factors like capsular polysaccharides, siderophores, aggregative adhesion, and both types 1 and 3 fimbriae play a major role in the severity level of* K. pneumoniae* infections [9].

Most researches are dedicated to studying either antimicrobial resistance or virulence, though the biological effect and relation between those factors are of particular importance. Since the third-generation cephalosporins, like other *β*-lactam antibiotics, are crucial for treatment of severe hospital-onset or community-acquired infections caused by* K. pneumoniae* [10], therefore, studying of both processes might provide better understanding of the relationship between *β*-lactam resistance and virulence.

Accordingly, this study aims to gain further insight into virulence characteristics of ESBLs and non-ESBLs producing* K. pneumoniae* isolates from Mansoura Hospitals. In addition, we sought to explore the genetic relatedness between ESBLs and non-ESBLs producing* K. pneumoniae.*

## 2. Materials and Methods

### 2.1. Bacterial Isolation and Identification

Hundred* K. pneumoniae* isolates were isolated from 243 clinical specimens. These clinical specimens were obtained from various clinical sources including sputum, urine, wounds, and burns at Mansoura Hospitals. All isolates were biochemically identified according to biochemical standards [11]. The protocol conducted in the study complies with the ethical guidelines and use and handling of human subjects in medical research adopted by The Research Ethics Committee, Faculty of Pharmacy, Mansoura University, Egypt (Permit Number: 2013-30).

### 2.2. Antimicrobial Sensitivity Testing

For each pure isolate, an antimicrobial sensitivity testing was performed by disk diffusion technique as described in the guidelines of the Clinical and Laboratory Standard Institute (2014) [12]. The following antibiotics were used: aztreonam (30 *μ*g), ceftriaxone (30 *μ*g), ceftazidime (30 *μ*g), cefotaxime (30 *μ*g), cefoperazone (30 *μ*g), and cefepime (30 *μ*g).

### 2.3. Detection of ESBL Producing Isolates

*K. pneumoniae* strains were initially tested for *β*-lactamase production according to Hassan et al. 2010 [13]. All positive *β*-lactamase-producing strains were subjected to the Modified Double Disc Synergy Test (MDDST) in order to determine the production of ESBL [14, 15]. ESBL production is inferred by any distortion or augmentation ≥5 mm of an inhibition zone of the cephalosporin discs towards the amoxicillin-clavulanate disc.

### 2.4. Detection of Virulence Factors of* K. pneumoniae* Isolates

#### 2.4.1. Blood Hemolysis

The plate hemolysis test was performed by streaking the isolates on blood agar plates which contain 5% (vol/vol) human blood. Total (*β*) and partial (*α*) red blood cell lysis were carefully detected after 24 hrs of incubation at 37°C [16].

#### 2.4.2. Haemagglutination

A slide method was adapted for detection of erythrocytes clumping by bacterial fimbriae as described by Vagarali et al. 2008 [17]. The test was done using human blood (type “O”). After three times of washing steps of red blood cells with saline, 3% RBCs suspension in fresh saline was prepared. A drop of this suspension was added to one drop of the tested bacterial culture. Then the slide was rolled for 5 min at room temperature. Clumping was considered as a positive haemagglutination result.

#### 2.4.3. Serum Resistance

Serum resistance was analyzed using the turbidimetric assay. The absorbance at 620 nm was carefully measured before and after three hours of incubation at 37°C. The average of 2 replicates was accepted to determine the final absorbance, and the mean of remaining absorbance relative to the initial absorbance before incubation was calculated. If the ratio was higher than 100%, the isolates were considered serum resistant [18].

#### 2.4.4. Biofilm Detection

The ability of bacteria to form biofilm was assessed using microtiter plate assay [19, 20]. For each isolate, the mean OD_492_ of the six wells was calculated (OD_T_). The cut-off OD (ODc) was defined as three standard deviations above the mean OD of the negative control wells. The level of the formed biofilm was asserted as follows:

- Nonadherent:  OD_*T*_ ≤ OD_*C*_
- Weakly adherent:  OD_*C*_ < OD_*T*_ ≤ 2OD_*C*_
- Moderately adherent:  2OD_*C*_ < OD_*T*_ ≤ 4OD_*C*_
- Strongly adherent:  4OD_*C*_ < OD_*T*_.

#### 2.4.5. Lipase Production

According to Panus et al. 2008 [16], isolates were streaked individually on tween 80 agar (1%). After a week of incubation at 37°C, lipase producing isolates form an opaque precipitation zones.

#### 2.4.6. Phenotypic Detection of Hypermucoviscosity (HMV)

It was done using a modified string test in which single colonies were tested for their ability to stretch a mucoviscous string. When the formed string stretched >10 mm in length, it indicated HMV phenotype [21].

#### 2.4.7. Gelatinase Production

The production of gelatinase was identified after streaking bacteria in gelatin agar plates and incubation at 37°C for 24 hs. The gelatinase producing colonies were surrounded by a clear zone once mercuric chloride was poured on plates while the medium became opaque [22].

### 2.5. Polymerase Chain Analysis of Resistance and Virulence Genes

One single colony of each isolate was suspended in 70 *μ*l DNase-free water and subjected to heat block at 95°C for 10 min. The ESBLs genes (*TEM, SHV, *and* CTX-M-15*) and virulence genes including* fim H* for haemagglutination,* BssS* for biofilm formation,* iss* and* traT* for serum resistance gene, and* iucA* for aerobactin gene were amplified using Dream Taq PCR Master Mix (Fermentas, US) and primers listed in Table 1. The reaction mixture composed of 12.5 *μ*l Dream Taq Green PCR Master Mix (2x), 1 *μ*l of forward primer (10 *μ*M), 1 *μ*l of reverse primer (10 *μ*M), 1 *μ*l of bacterial lysate, and 9.5 *μ*l of nuclease-free water which were added for a total of 25 *μ*l per reaction. A negative PCR control was prepared. The cycling conditions started with initial denaturing at 95°C for 5 min, followed by 40 cycles of denaturation at 95°C for 30 s, annealing for 30 s at temperatures specified for each primer as listed in Table 1, and extension at 72°C for 1 min. This was followed by a final extension step at 72°C for 5 mins.

### 2.6. Random Amplified Polymorphic DNA (RAPD) Profile

According to Rodrigues et al. 2008, the primer Operon 18 (5′-CAGCACCCAC-3′) was used to generate suitable RAPD banding profiles [25]. RAPD was performed according to the method of Eftekhar and Nouri, 2015, with some modification [26]. The reaction mixtures (20 ml) contained 1 *μ*M of the used primer, 0.2 mM dNTP, 1.5 U of FlexiTaq DNA polymerase, 1x GoTaq® Flexi buffer, 0.5 mM MgCl_2_, and 3 *μ*l of DNA template. RAPD-PCR was performed in a thermal cycler (FPROGO2D, Techne Ltd., Cambridge, UK) using the following program: initial denaturation at 95°C for 3 min followed by 40 cycles of denaturation for 30 sec at 95°C, annealing for 30 sec at 37°C and extension for 2 min at 72°C, and then a final extension step at 72°C for 10 min. The amplified products were visualized by UV transillumination after electrophoresis on 1% agarose gel stained with ethidium bromide. RAPD fingerprints were analyzed by visual inspection and compared with a 100 bp plus DNA molecular weight ladder.

### 2.7. Statistical Analysis

Data representing the presence of different virulence factors associated genes in both groups, the ESBL and non-ESBL, were analyzed by performing the *x*^2^ test or Fisher exact test. The significance of differences was evaluated at *P* ≤ 0.05.

## 3. Results

### 3.1. Bacterial Isolation and Identification

Two hundred and forty-three clinical isolates were collected from different patients in Mansoura Hospitals, Egypt. Hundred isolates were purified and identified biochemically as* K. pneumoniae*. The majority of* K. pneumoniae* isolates were obtained from urine (74%), sputum (11%), wounds (9%), and burns (6%).

### 3.2. Antimicrobial Sensitivity Testing of* K. pneumoniae* Isolates

The antimicrobial sensitivity pattern of* K. pneumoniae* isolates was determined by disc diffusion method. Forty-nine isolates (49%) were resistant to ceftriaxone and cefotaxime. Fifty isolates (50%) were resistant to cefoperazone. Regarding ceftazidime and aztreonam, it was found that 40 (40%) and 38 (38%) isolates were resistance to both antibiotics, respectively. On the other hand, only 19 (19%) of the isolated* K. pneumoniae* were resistant to cefepime.

### 3.3. Detection of Extended Spectrum *β*-Lactamase (ESBL) Producing Isolates

Detection of ESBL revealed that 50% of the tested isolates were ESBL producers and all these isolates harbored at least two ESBL genes (*SHV*,* TEM,* or* CTX-M*-*15*) (Table 2). Moreover, ESBL producers exhibited a significant decreased susceptibility to all the tested beta-lactams compared with non-ESBL producers (*P* < 0.0001).

### 3.4. Phenotypic and Genotypic Detection of Virulence Factors

The virulence features of 50 ESBLs and 50 non-ESBLs producing isolates are shown in Tables 2 and 3, respectively.

The blood hemolysis test for all isolates revealed that only one ESBL and two non-ESBL producing isolates were *α*-hemolytic.

All isolates were tested for their ability to agglutinate erythrocytes. Clumping of erythrocytes was observed in 48 ESBL producing isolates (96%) and 47 non-ESBL producing isolates (94%). PCR detection of* fim H* gene revealed that all ESBL and non-ESBL producing isolates harbored* fim H* gene.

Serum resistance of all isolates was analyzed using a turbidimetric assay. The remaining absorbance after 3 hours (OD_620_, 3 h) was greater than 100% relative to the initial absorbance in 29 (58%) of ESBL isolates and in 11 (22%) of non-ESBL isolates, so these isolates were designated serum resistant and the difference was highly significant (*P* < 0.0001). The remaining isolates showed sensitivity to serum. PCR analysis revealed that none of the tested isolates harbored* traT* gene. In contrast,* iss* gene was detected in 50% and 22% of ESBL and non-ESBL isolates, respectively (*P* < 0.0001).

**Figure 1:**
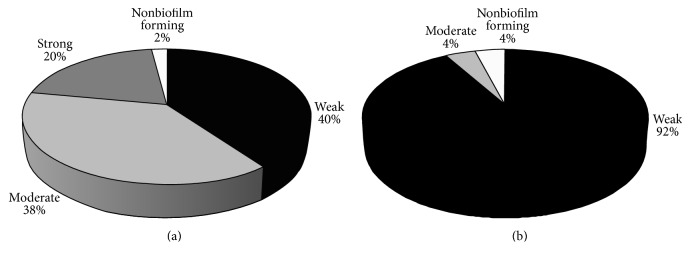
Categories of biofilm intensity of ESBLs and non-ESBLs producing* K. pneumoniae*. (a) ESBLs producers; (b) non-ESBLs producers.

Biofilm formation of all isolates was tested using microtiter plate assay. Biofilm intensity was classified as weak, moderate, and strong and was compared among ESBL and non-ESBL producers (Figure 1). Weak biofilm was detected in 40% of ESBL producers and 92% of non-ESBL with highly significant difference (*P* < 0.0001). Moderate type of biofilm was higher in ESBL (38%) compared to non-ESBLs (4%) (*P* < 0.0001). Moreover, strong biofilm production was detected only among ESBL producers (20%) (*P* < 0.0001). Only one ESBL producer and 2 non-ESBL producers were nonbiofilm producers. Regarding* BssS* gene, it was found among all isolates.

For lipase production only 3 (6%) ESBL and 5 (10%) non-ESBL producing isolates were considered lipase producers with no significant difference of both groups.

The prevalence of HV phenotype was higher among non-ESBLs producing isolates where 31 (62%) of non-ESBLs exhibited hypermucoviscosity compared to the ESBLs (4%) (*P* < 0.0001).

No significance difference was observed between ESBL and non-ESBL producing isolates in gelatinase production.

Aerobactin gene (*iucA*) was detected in 6 (12%) of ESBLs and 3 (6%) of non-ESBL producing isolates.

### 3.5. Virulence Profiles Associated with ESBLs and Non-ESBLs Producing Isolates

A total of twenty-four different virulence profiles were observed among the tested isolates. Six profiles were associated with ESBLs producing isolates compared to ten profiles for non-ESBLs producing isolates. In addition, seven profiles were found in both types of isolates. The most prevalent profiles associated with ESBLs producing isolates were biofilm-serum resistant-haemagglutination-*BssS-fimH*-*iss* (28%), while the most common profiles observed with non-ESBLs producing isolates were biofilm-haemagglutination-hypermucoviscosity-*BssS-fimH* (36%) (Table 4).

### 3.6. RAPD Profile Analysis

All isolates were typed by RAPD-PCR analysis. The number of patterns generated by operon 18 was 51 as shown in Tables 2 and 3. Eighteen patterns were specific for ESBL producing isolates, 32 patterns were specific for non-ESBL producing isolates, and 1 pattern (P8) was exhibited by both types of isolates. Of the eighteen RAPD patterns associated with ESBLs producing isolates, P3 was the most predominant (14%). The second most common pattern was P2; it was observed among 12% of ESBLs producing isolates. In addition, eight patterns were represented by single isolate. Overall, non-ESBL producing isolates were more diverse than ESBL producing isolates, where 26 out of 32 patterns were represented by single isolate.

**Figure 2:**
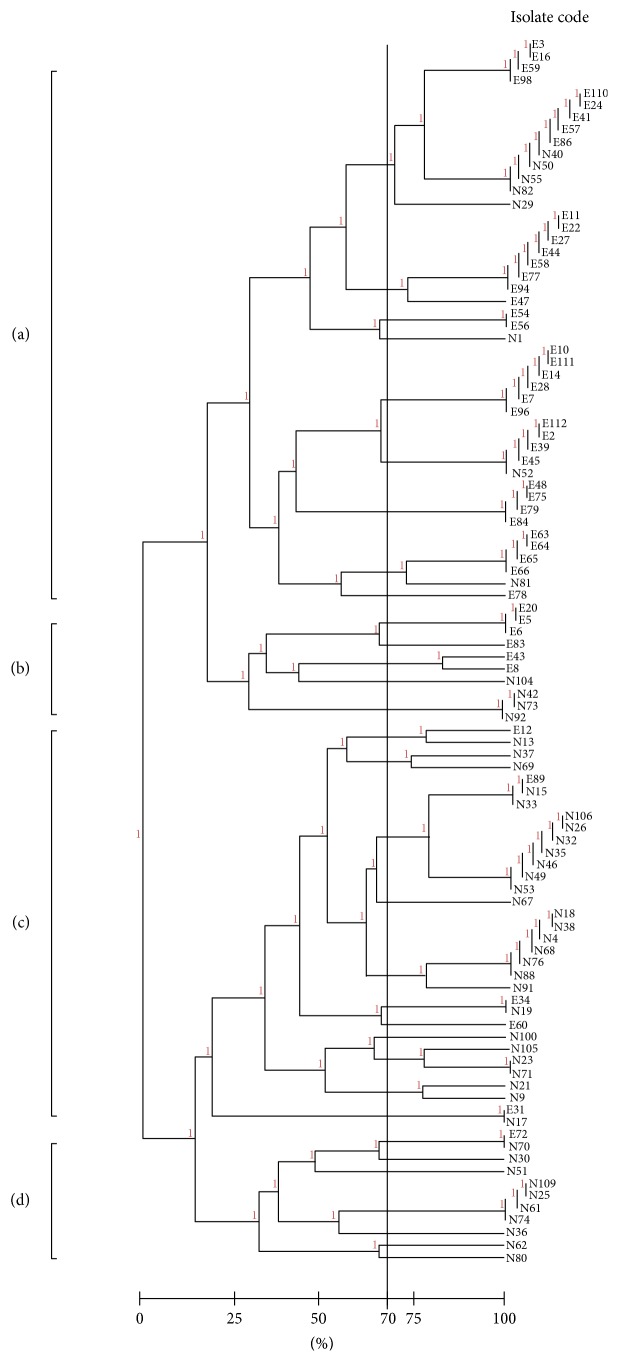
Dendrogram of RAPD-operon 18 profile of the 100 ESBLs and non-ESBLs producing* K. pneumoniae*.** (**a) Cluster a, (b) cluster b,** (**c) cluster c, and (d) cluster d.

Cluster analysis of RAPD profile classified all isolates into four clusters a, b, c, and d (Figure 2). The four groups consisted of both ESBLs and non-ESBL producing isolates with different level of distribution. ESBLs producing isolates were the most dispersed in cluster a (76%) (*n* = 38) compared to non-ESBL producing isolates (16%) (*n* = 8). 12% (*n* = 6) of ESBLs producing isolates were identified in cluster b while 8% (*n* = 4) of non-ESBLs producing isolates were present in the same group. In contrast non-ESBLs producing isolates were more predominant in clusters c and d where 56% (*n* = 28) and 20% (*n* = 10) of these isolates were included in both groups, respectively. ESBLs producing isolates comprised lower percent in both clusters c and d.

## 4. Discussion

*Klebsiella pneumoniae *is a common pathogen associated with both community and hospital-acquired infections including respiratory and urinary tract infections and wound and blood infections [27]. Its pathogenicity is related to a multitude of virulence factors [28] and ability to readily acquire multiple antibiotic resistances [29]. In fact, it is an important host of ESBL. Bacterial resistance to *β*-lactams by ESBL production has increased dramatically in human pathogens, causing significant morbidity and mortality [30].

The proportion of* K. pneumoniae* isolates producing ESBL is variable among countries. These proportions were 12% in the United States, 33% in Europe, 52% in Latin America, and 28% in the Western Pacific [31]. In the study of Shin and Ko, 2014, 33.6% of the isolates were ESBLs producer [32]. A higher percent was found in Arabian region where Aljanaby and Alhasani, 2016, reported that rate of ESBL producing* K. pneumoniae* was 62.5% in AL-Najaf Governorate, Iraq [33]. In this study, 50% of isolates were estimated as ESBLs producers. These data confirm the dramatic spread of ESBL isolates all over the world.

Infections resulting from ESBL producers are associated with serious adverse conditions [34]. Indeed, this is related to both ineffective therapy and the failure in the choice of an antibiotic active against these isolates. However, the increased incidence of mortality associated with ESBL producers may also be associated with the increasing virulence of these isolates [35].

Most *β*-lactamases contribute to resistance to a variety of antibiotics including the third- and fourth-generation cephalosporins and monobactams [36]. This study confirms that ESBL producing isolates exhibited significantly greater resistance to the examined beta-lactams than did non-ESBL producers (*P* < 0.0001). These results are comparable to that previously reported by Shin and Ko, 2014, where ESBL producing isolates showed a significant higher resistance to most beta-lactams than did non-ESBL producing isolates (*P* < 0.05) [32].

*K. pneumoniae* mostly harbors ESBL genes (*SHV*,* TEM,* and* CTX-M*) which have shown resistance to the majority of antibiotics [37]. In this study, PCR detection of these genes revealed that 100, 96, and 84% of ESBL producing* K. pneumoniae* harbored C*TX-M-*_*15*_,* SHV,* and* TEM,* respectively (Table 2). Shin and Ko, 2014, showed similar results regarding* CTX-M* where all ESBL producers were found to harbor bla*CTX-M *gene [33]. Additionally, Aljanaby and Alhasani, 2016, reported that* TEM* and* SHV* were 93.75% (30/32) and 87.5% (28/32), respectively [33].

The pathogenicity of* K. pneumoniae* is a result of a variety of virulence factors that cause multiple diseases through attacking the immune system of mammalians [23]. Infections caused by ESBL producing* K. pneumoniae* are linked to severe conditions due to the capability of these strains to express virulence factors [35]. Microbial biofilm formation and development have been reported to have major role in* Klebsiella* pathogenicity. In addition, biofilms can protect bacteria from exposure to antimicrobials when compared with other nonbiofilm forming bacteria [38]. In the current study, biofilm was highly prevalent in both ESBL and non-ESBL producers. More importantly, development of strong and moderate biofilm is much more significant in ESBL producers compared to the non-ESBLs (Figure 1). Type 1 or type 3 fimbriae are the most important virulence factors responsible for adhesion of* K. pneumoniae *and increasing its ability to grow in biofilm community [9]. This explains why* fimH* gene was found in all biofilm producing isolates.

Serum resistance has been shown in multiple bacterial systems to be critical for the survival of invading bacteria and the establishments of disease, since mutations resulting in loss of serum resistance render several bacterial pathogens avirulent [39]. Because serum resistance is one of the pathogenicity factors of* Klebsiella*, the superior resistance to serum bactericidal activity in the present study (40%) is an indicator of their higher pathogenicity. Gundogan and Yakar, 2007, found that 32.5% of* K. pneumoniae* were serum resistant [40]. There are several studies reporting that there was positive association between ESBL and serum resistance. In the present study, comparing serum resistance among our tested isolates, ESBLs producers were significantly higher serum resistant than did non-ESBL producers. This result is matching with that previously established by Sahly et al. 2004 [41] which revealed that the prevalence of serum resistant isolates was greatly observed among ESBL producing isolates (*TEM* and* SHV* types) (30%; 27/90 isolates) compared to non-ESBL producers (17.9%; 32/178 isolates) (*P* = 0.037). Lin et al., 2016, reported that the percentage of serum resistance was significantly higher among the ESBL producing* K. pneumoniae* strains than among the non-ESBL producing* K. pneumoniae* strains [42].

Gene* traT* was not detected among the tested isolates. In a previous study of Atmani et al., 2015 [30]* traT* gene was present at low rate (3.1%) in municipal wastewater-treatment plant isolates and was absent in hospital effluents and clinical isolates. This serum resistance-associated outer membrane plasmid gene was previously reported in clinical isolates as minor contributor in serum resistance [23].

In the present study* iss* gene was detected in 50% and 22% of ESBL and non-ESBL producing isolates, respectively (*P* < 0.0001). In the study of El-Mahdy et al., 2011* iss* gene was found in 32% on genomic DNA and in 36% on plasmid DNA of* E. coli* isolates. In the same study* iss* gene was detected in 5% on genomic DNA and in 31% on plasmid DNA of* K. pneumoniae* isolates [43]. This confirms the horizontal transfer of* iss* gene among bacteria. Our finding that* iss* gene was detected in 65% of serum resistant isolates suggests that this gene might be related to serum resistance (Tables 2 and 3).

Diverse capsular ingredient and an increased amount of capsular material have been described in hypervirulent* K. pneumoniae* isolates [44]. However, little work elucidating the role of the hypermucoviscous (HMV) phenotype in the pathogenicity of* K. pneumoniae* exists, and no direct comparison of HMV and non-HMV isolates using the innate immune system components of susceptible hosts has been described. This is because a number of genetic loci appear to be related to the HMV phenotype of* K. pneumoniae* [45].

In the study of Lee et al., 2016, 94.3% of the isolates expressed the hypermucoviscous phenotype (capsular type K1/K2/K5) and they were serum resistant. In addition, 57.1% of nonhypermucoviscous (non-K1/K2/K5) isolates were also serum resistant. Lee et al., 2016, confirmed that hypermucoviscosity and serum resistance phenomena depend on the type of capsule [46]. In addition, Fang et al., 2004, found that high serum resistance was detected among eight randomly selected clinical* K. pneumoniae* isolates: four of them were HMV invasive isolates and four were non-HV noninvasive isolates [47]. Moreover, El Fertas-Aissani et al., 2013, reported that the hypermucoviscosity was found only in 9.2% of isolates although 92.6% of the isolates were serum resistant [23].

In the present study hypermucoviscosity was estimated among 33% of* K. pneumoniae* isolates. It was found that 8 (24%) HMV isolates exhibited serum resistance. Previous reports have indicated that ESBL genes are rarely detected in* K. pneumoniae* strains with the HMV phenotype and there is also negative association between hypermucoviscosity (HV) and ESBL [48]. This finding is supported by the result of our study where 62% of non-ESBLs exhibited hypermucoviscosity compared to the ESBLs (4%) (*P* < 0.0001). In the study of Lee et al., 2010, HMV phenotypes were identified in 35 (38.5%) of 91* K. pneumoniae* isolates. Detection of ESBLs in the same study revealed that 24 isolates (26.4%) were ESBL producing strains. Only one ESBL producing* K. pneumoniae* strain expressed the HMV phenotype. Their results indicated a significant negative association between the HMV phenotype and ESBL production in* K. pneumoniae* isolates [48]. Moreover, Yu et al., 2015, confirmed that the prevalence of the HMV phenotype was significantly lower in ESBL* K. pneumoniae* isolates (8.8%) than that in non-ESBL* K. pneumoniae* isolates (53.8%) [49].

Aerobactin is a citrate-hydroxamate siderophore rarely expressed by classical nosocomial* K. pneumoniae*. It is more expressed in HMV* K. pneumoniae* [50]. However, in our study 15.1% of HMV* K. pneumoniae *were aerobactin producers. These results were relatively similar to that reported by El Fertas-Aissani et al., 2013, where only 20% of HMV were aerobactin producers [23]. Furthermore, this siderophore is not common among both ESBL and non-ESBL producers in this study and as previously reported by Podschun et al. 2001 [51] and Atmani et al. 2015 [30]. Moreover, *α*-hemolysis which is often associated with virulence of various pathogenic microorganisms was very rare among our isolates (2% in ESBL and 4% in non-ESBL producers) and previous studies [32, 52]. However, Gundogan et al., 2011, [53] have confirmed that 67% of* Klebsiella* isolates from meat samples exhibited hemolytic activity. Likewise, both gelatinase and lipase enzymes are minor contributors of virulence in both ESBL and non-ESBL producers.

Analysis of virulence factors combination has brought out 23 different virulence profiles including 2 to 8 virulence factors (Table 4). Thirteen profiles were observed among ESBL producers and seventeen among non-ESBL producing isolates, of which seven profiles were shared by both isolates. Indeed, four of the established virulence profiles were circulated among 76% of ESBL producing isolates. The remaining nine virulence profiles of ESBL producers included one, two, or three isolates for each profile. Regarding non-ESBL producers, two virulence profiles were detected among 60% of the isolates. The remaining fifteen virulence profiles of non-ESBL producers included from one to three isolates in each profile. These findings suggest that ESBL producers were more genetically related than non-ESBL producers. Our observation was confirmed by RAPD analysis (Tables 2 and 3). This technique has been commonly used as an epidemiological tool to differentiate between different* K. pneumoniae* isolates [54]. Overall, our results confirmed a marked genetic relatedness among ESBL compared to the non-ESBL producers where eight out of eighteen RAPD patterns specific for ESBL were represented by single isolate, while 26 out of 32 RAPD patterns specific for non-ESBL were represented by single isolate. Dendogram analysis of RAPD profile classified all isolates into four clusters (a, b, c, and d) based on numerous fingerprints generated (Figure 2). The majority of ESBL isolates (*n* = 38, 76%) belonged to group a which in turn could confirm the genetic relatedness among ESBL producing isolates. In contrast, non-ESBL producers were genetically diverse where 16%, 8%, and 20% of the isolates were distributed among clusters a, b, and d, respectively, although half of nonproducing isolates were in cluster c (*n* = 28, 56%). On contrast, Eftekhar and Nouri 2015 [26] reported that most non-ESBL isolates (62.1%) belonged to a single cluster and the ESBL producers and their RAPD fingerprints were spread among 8 clusters.

In conclusion, this is the first study conducted in Mansoura University that shows the differences in virulence characteristics between ESBLs and non-ESBLs producing* K. pneumoniae.* Accordingly, this study suggests a correlation between ESBL production and some virulence factors. Therefore increased alertness of clinicians and enhanced testing by laboratories are necessary to reduce failure of therapy and prevent the dissemination of ESBL producing* K. pneumoniae*.

## Figures and Tables

**Table 1:** Oligonucleotide primers used for extended spectrum *β*-lactamase and virulence gene detection.

Gene name	Type	Primer Sequence	Annealing temp.	Amplicon size (bp)^c^	Reference
*SHV*	Fw^a^	5′-ACTATCGCCAGCAGGATC-3′	53°C	356	This study
	Rev^b^	5′-ATCGTCCACCATCCACTG-3′			
*TEM*	Fw	5′-GATCTCAACAGCGGTAAG-3′	50°C	786	This study
	Rev	5′-CAGTGAGGCACCTATCTC-3′			
*CTX-M-*_*15*_	Fw	5′-GTGATACCACTTCACCTC-3′	49°C	255	This study
	Rev	5′-AGTAAGTGACCAGAATCAG-3′			
*TraT*	Fw	5′-GGTGTGGTGCGATGAGCACAG-3′	63°C	290	[23]
	Rev	5′-CACGGTTCAGCCATCCCTGAG-3′			
*FimH*	Fw	5′-TACTGCTGATGGGCTGGTC-3′	50°C	640	[24]
	Rev	5′-GCCGGAGAGGTAATACCCC-3′			
*Iss*	Fw	5′-GGCAATGCTTATTACAGGATGTGC-3′	50°C	260	[24]
	Rev	5′-GAGCAATATACCCGGGCTTCC-3′			
*BssS*	Fw	5′-GATTCAATTTTGGCGATTCCTGC-3′	48°C	225	[24]
	Rev	5′-TAATGAAGTCATTCAGACTCATCC-3′			
*iucA*	Fw	5′-CGAAATCGAAATAGATCACC-3′	51°C	1125	[24]
	Rev	5′-CTGACGCGATTTGCCGC-3′			

^a^Forward, ^b^reverse, and ^c^base pair.

**Table 2:** Clinical data, RAPD, positive virulence characteristics, and *β*-lactamase characteristics of ESBL producing *K. pneumoniae* isolates.

Isolate code	Clinical source	Hosp^c^	Virulence characteristics	RAPD^a^ profile										RAPD p^b^	ESBL type
E2	Urine	UNC^d^	Biofilm, haem^g^*, BssS*, *fimH*				|		|	|				P5	S^h^, C^i^
E3	Urine	UNC	Biofilm, haem, *BssS, fimH*			|	|	|	|	|	|			P1	S, T^j^, C
E5	Urine	UNC	Biofilm, haem, hypermucoviscosity, *BssS, fimH*, *iss*, *iucA*				|		|				|	P7	S, T, C
E6	Wound	MUH^e^	Biofilm, haem,* BssS, fimH*, *iss*				|		|				|	P7	S, T, C
E7	Urine	UNC	Biofilm, serum resistant, haem, *BssS, fimH*			|	|		|	|				P2	S, T, C
E8	Urine	UNC	Biofilm, serum resistant, haem, *BssS, fimH*, *iss*		|	|	|	|	|	|		|	|	P17	S, T, C
E10	Urine	UNC	Biofilm, haem, *BssS, fimH*, *iss*			|	|		|	|				P2	S, T, C
E11	Urine	UNC	Biofilm, serum resistant, haem, *BssS, fimH*, *iss*			|	|		|		|			P3	S, T, C
E12	Urine	UNC	Biofilm, haem, *BssS, fimH*	|			|		|	|	|		|	P6	S, T, C
E14	Urine	UNC	Biofilm, serum resistant, haem, *BssS, fimH*			|	|		|	|				P2	S, T, C
E16	Sputum	CH^f^	Biofilm, *BssS, fimH*			|	|	|	|	|	|			P1	S, T, C
E20	Sputum	CH	Biofilm, haem, *BssS, fimH*, *iss*				|		|				|	P7	S, T, C
E22	Urine	UNC	Biofilm, haem, *BssS, fimH*			|	|		|		|			P3	S, T, C
E24	Urine	UNC	Biofilm, serum resistant, haem, * BssS, fimH*, *iss*			|	|		|	|	|			P4	S, C
E27	Urine	UNC	Biofilm, serum resistant, haem, *BssS, fimH*			|	|		|		|			P3	S, T, C
E28	Wound	MUH	Biofilm, haem, *BssS, fimH*			|	|		|	|				P2	S, C
E31	Burn	MUH	Biofilm, serum resistant, haem, lipase, *BssS, fimH*				|				|			P8	S, T, C
E34	Urine	UNC	Biofilm, serum resistant, haem, *BssS, fimH*, *iucA*, *iss*				|				|		|	P9	S, T, C
E39	Burn	MUH	Biofilm, haem, *BssS, fimH*				|		|	|				P5	S, T, C
E41	Urine	UNC	Biofilm, haem, *BssS, fimH*, *iss*			|	|		|	|	|			P4	S, T, C
E43	Urine	UNC	Biofilm, serum resistant, haem, *BssS, fimH, iucA*, *iss *			|	|	|	|	|	|	|	|	P10	T, C
E44	Urine	UNC	Biofilm, serum resistant, haem, lipase,* BssS,fimH*, *α*-hemolysis			|	|		|		|			P3	S, T, C
E45	Urine	UNC	Biofilm, serum resistant, haem, *BssS, fimH*, *iss*				|		|	|				P5	S, C
E47	Urine	UNC	Biofilm, serum resistant, haem,* BssS, fimH*			|	|	|	|		|			P11	S, T, C
E48	Urine	UNC	Biofilm, serum resistant, haem, *BssS, fimH*, *iucA*				|		|					P12	S, T, C
E54	Urine	UNC	Biofilm, serum resistant, haem, *BssS, fimH*, *iss*				|		|		|			P13	S, T, C
E56	Urine	UNC	Biofilm, serum resistant, haem, lipase, *BssS, fimH*				|		|		|			P13	S, T, C
E57	Urine	UNC	Biofilm, serum resistant, haem, *BssS, fimH*, *iss*			|	|		|	|	|			P4	S, T, C
E58	Urine	UNC	Biofilm, serum resistant, haem, *BssS, fimH*			|	|		|		|			P3	S, T, C
E59	Urine	UNC	Biofilm, serum resistant, haem, *BssS, fimH*, *iss*			|	|	|	|	|	|			P1	S, T, C
E60	Urine	UNC	Biofilm, serum resistant, haem, *BssS, fimH*, *iss*	|			|				|		|	P9	S, C
E63	Urine	UNC	Biofilm, serum resistant, haem, *BssS, fimH*			|	|		|				|	P14	S, T, C
E64	Urine	UNC	Biofilm, haem, *BssS, fimH*			|	|		|				|	P14	S, T, C
E65	Urine	UNC	Biofilm, serum resistant, haem, *BssS, fimH*, *iss*											P14	T, C
E66	Urine	UNC	Biofilm, haem, *BssS, fimH*, *iss*			|	|		|				|	P14	S, T, C
E72	Burn	MUH	Biofilm, haem, *BssS, fimH*				|			|	|			P15	S, T, C
E75	Burn	MUH	Biofilm, serum resistant, haem, *BssS, fimH*, *iss*				|		|					P12	S, C
E77	Urine	UNC	Biofilm, haem, *BssS, fimH*, *iss*			|	|		|		|			P3	S, T, C
E78	Sputum	C H	Biofilm, haem, *BssS, fimH*			|	|		|					P16	S, T, C
E79	Urine	UNC	Biofilm, serum resistant, haem, *BssS, fimH*, *iss*				|		|					P12	S, T, C
E83	Wound	MUH	*BssS, fimH*				|		|			|	|	P18	S, C
E84	Urine	UNC	Biofilm, serum resistant, haem, *BssS, fimH*, *iss*				|		|					P12	S, T, C
E86	Urine	UNC	Biofilm, serum resistant, haem, *BssS, fimH*			|	|		|	|	|			P4	S, T, C
E89	Urine	UNC	Biofilm, haem, *BssS, fimH*			|	|		|	|	|		|	P19	S, C
E94	Urine	UNC	Biofilm, serum resistant, haem,* BssS,fimH*, *iss*			|	|		|		|			P3	S, T, C
E96	Urine	UNC	Biofilm, haem, *BssS, fimH*			|	|		|	|				P2	S, T, C
E98	Wound	MUH	Biofilm, serum resistant, haem,* BssS,fimH*, *iucA*, *iss*			|	|	|	|	|	|			P1	S, T, C
E110	Sputum	MUH	Biofilm, serum resistant, haem, hypermucoviscosity, *BssS, fimH*, *iucA*, *iss*			|	|		|	|	|			P4	S, T, C
E111	Urine	UNC	Biofilm, serum resistant, haem, *BssS, fimH*, *iss*			|	|		|	|				P2	S, T, C
E112	Urine	UNC	Biofilm, haem, gelatinase, *BssS, fimH*				|		|	|				P5	S, T, C

^a^Random amplified polymorphic DNA, ^b^pattern, ^c^hospital, ^d^Urology and Nephrology Center, ^e^Mansoura University Hospital, ^f^Chest Hospital, ^g^haemagglutination, ^h^*SHV*, ^i^*CTX-M-*_15_, and ^j^*TEM*.

**Table 3:** Clinical data, RAPD, and positive virulence characteristics of non-ESBL producing *K. pneumoniae* isolates.

Isolate code	Clinical source	Hosp^c^	Virulence characteristics	RAPD^a^ profile											RAPD p^b^
N1	Urine	UNC^d^	Hyper mucoviscosity, *BssS, fimH*				|		|	|		|			P20
N4	Sputum	MUH^e^	Biofilm, haem^g^, hypermucoviscosity, *BssS, fimH*				|		|	|		|		|	P28
N9	Urine	MUH	Biofilm, haem, *BssS, fimH*		|	|	|			|		|		|	P50
N13	Urine	UNC	Biofilm, haem, hypermucoviscosity, *BssS, fimH*	|			|			|		|		|	P26
N15	Urine	UNC	Biofilm, haem, *BssS, fimH*			|	|		|	|		|		|	P27
N17	Urine	UNC	Biofilm, haem, *BssS, fimH*, *iss*				|					|			P8
N18	Urine	UNC	Biofilm, haem, hypermucoviscosity, *BssS, fimH*				|		|	|		|		|	P28
N19	Sputum	CH^f^	Biofilm, haem, hypermucoviscosity, *BssS, fimH*				|					|		|	P29
N21	Urine	UNC	Biofilm, haem, *α*-hemolysis, hyper mucoviscosity, *BssS, fimH*		|	|	|			|				|	P30
N23	Urine	UNC	Biofilm, serum resistant, haem, hyper mucoviscosity, *BssS, fimH*, *iss*, *iucA*		|		|			|		|		|	P31
N25	Sputum	MUH	Biofilm, haem, hypermucoviscosity, *BssS, fimH*				|			|					P25
N26	Urine	UNC	Biofilm, serum resistant, haem, *α*-hemolysis, lipase, hypermucoviscosity, *BssS, fimH*, *iss*			|	|		|			|		|	P32
N29	Wound	MUH	Biofilm, haem*, BssS, fimH*			|	|		|	|		|	|		P33
N30	Wound	MUH	Biofilm, *BssS, fimH*		|		|			|		|			P34
N32	Wound	MUH	Biofilm, haem, gelatinase, hypermucoviscosity, *BssS, fimH*			|	|		|			|		|	P32
N33	Urine	UNC	Biofilm, serum resistant, haem, hypermucoviscosity, *BssS, fimH*, *iss*			|	|		|	|		|		|	P35
N35	Urine	UNC	Biofilm, serum resistant, haem, *BssS, fimH*, *iss*			|	|		|			|		|	P32
N36	Urine	UNC	Biofilm, haem, hypermucoviscosity, *BssS, fimH*				|			|				|	P36
N37	Urine	UNC	Biofilm, haem, *BssS, fimH*				|			|		|		|	P37
N38	Urine	UNC	Biofilm, haem, hypermucoviscosity, *BssS, fimH*				|		|	|		|		|	P28
N40	Urine	UNC	Biofilm, haem, lipase, hypermucoviscosity, *BssS, fimH, iucA*, *iss*			|	|		|	|		|			P38
N42	Urine	UNC	Biofilm, haem, hypermucoviscosity, *BssS, fimH*				|	|	|				|		P39
N46	Urine	UNC	Biofilm, haem, hypermucoviscosity, *BssS, fimH*			|	|		|			|		|	P32
N49	Urine	UNC	Hypermucoviscosity, *BssS, fimH*			|	|		|			|		|	P32
N50	Urine	UNC	Biofilm, haem, hypermucoviscosity, *BssS, fimH*			|	|		|	|		|			P40
N51	Urine	UNC	Biofilm, haem, *BssS, fimH*		|		|			|					P41
N52	Urine	UNC	Biofilm, haem, gelatinase, *BssS, fimH*				|		|	|					P42
N53	Urine	UNC	Biofilm, serum resistant, haem, *BssS, fimH*, *iss*			|	|		|			|		|	P32
N55	Urine	UNC	Biofilm, haem,* BssS, fimH*			|	|		|	|		|			P40
N61	Urine	UNC	Biofilm, haem, hypermucoviscosity, *BssS, fimH*				|			|					P25
N62	Urine	UNC	Biofilm, haem, hypermucoviscosity, *BssS, fimH*			|	|			|					P43
N67	Wound	MUH	Biofilm, haem, *BssS, fimH*				|		|			|		|	P44
N68	Urine	UNC	Biofilm, haem, hypermucoviscosity, *BssS, fimH*				|		|	|		|		|	P28
N69	Urine	UNC	Biofilm, haem, hypermucoviscosity, *BssS, fimH*			|	|			|		|		|	P45
N70	Urine	UNC	Biofilm, haem, *BssS, fimH*				|			|		|			P46
N71	Sputum	MUH	Biofilm, haem, hypermucoviscosity, *BssS, fimH*		|		|			|		|		|	P31
N73	Urine	MUH	Biofilm, haem, *BssS, fimH*				|	|	|				|		P39
N74	Sputum	MUH	Biofilm, haem, hypermucoviscosity, *BssS, fimH*				|			|					P25
N76	Urine	UNC	Biofilm, serum resistant, haem, hypermucoviscosity, *BssS, fimH*, *iss*				|		|	|		|		|	P47
N80	Wound	MUH	Biofilm, haem*, BssS, fimH*			|	|			|		|			P48
N81	Burn	MUH	Biofilm, haem, *BssS, fimH*			|	|	|	|					|	P49
N82	Urine	MUH	Biofilm, haem, *BssS, fimH*			|	|		|	|		|			P40
N88	Urine	MUH	Biofilm, serum resistant, haem, *BssS, fimH*, *iss*				|		|	|		|		|	P28
N91	Urine	UNC	Biofilm, serum resistant, haem, *BssS, fimH*		|		|		|	|		|		|	P51
N92	Urine	UNC	Biofilm, serum resistant, haem, lipase, hypermucoviscosity, *BssS, fimH*				|	|	|				|		P39
N100	Urine	UNC	Biofilm, serum resistant, haem, lipase, hypermucoviscosity, *BssS, fimH*, *iss*		|		|					|		|	P21
N104	Burn	MUH	Biofilm, haem, hypermucoviscosity, *BssS, fimH*			|	|		|		|		|	|	P22
N105	Urine	UNC	Biofilm, serum resistant, haem, lipase, hypermucoviscosity, *BssS, fimH*		|		|			|		|	|	|	P23
N106	Sputum	MUH	Biofilm, haem, hypermucoviscosity,* BssS, fimH*, *iucA*, *iss*			|	|		|			|		|	P24
N109	Sputum	MUH	Biofilm, haem, hypermucoviscosity, *BssS, fimH*				|			|					P25

^a^Random amplified polymorphic DNA, ^b^pattern, ^c^hospital, ^d^Urology and Nephrology Center, ^e^Mansoura University Hospital, ^f^Chest Hospital, and ^g^haemagglutination.

**Table 4:** Virulence profiles associated with ESBLs and non-ESBLs producing *K. pneumoniae i*solates.

Virulence profiles	ESBLs producing isolates number (%)	Non-ESBLs producing isolates number (%)
Biofilm-haemagglutination-*BssS-fimH*	11 (22%)	12 (24%)
Biofilm-haemagglutination-hypermucoviscosity-*BssS-fimH*-*iss-iucA*	1 (2%)	1 (2%)
Biofilm-haemagglutination-*BssS-fimH*-*iss*	6 (12%)	1 (2%)
Biofilm-serum resistant-haemagglutination-*BssS-fimH*	7 (14%)	1 (2%)
Biofilm-serum resistant-haemagglutination-*BssS-fimH*-*iss*	14 (28%)	3 (6%)
Biofilm-*BssS-fimH*	1 (2%)	1 (2%)
Biofilm-serum resistant-haemagglutination-lipase-*BssS-fimH*	2 (4%)	0 (0%)
Biofilm-serum resistant-haemagglutination-*BssS-fimH*-*iss-iucA*	3 (6%)	0 (0%)
Biofilm-serum resistant-haemagglutination-lipase-*α*-hemolysis*-BssS-fimH*	1 (2%)	0 (0%)
Biofilm-serum resistant-haemagglutination-*BssS-fimH-iucA*	1 (2%)	0 (0%)
*BssS-fimH*	1 (2%)	0 (0%)
Biofilm-serum resistant-haemagglutination*-*hypermucoviscosity*-BssS-fimH*-*iss-iucA*	1 (2%)	0 (0%)
Biofilm-haemagglutination-gelatinase-*BssS-fimH*	1 (2%)	1 (2%)
Hypermucoviscosity*-BssS-fimH*	0 (0%)	2 (4%)
Biofilm-haemagglutination-hypermucoviscosity-*BssS-fimH*	0 (0%)	18 (36%)
Biofilm-haemagglutination-hypermucoviscosity-*α*-hemolysis-*BssS-fimH*	0 (0%)	1 (2%)
Biofilm-serum resistant-haemagglutination-hypermucoviscosity-*BssS-fimH*-*iss*-*iucA*	0 (0%)	1 (2%)
Biofilm-serum resistant-haemagglutination-lipase-*α*-hemolysis*-BssS-fimH*-*iss*	0 (0%)	1 (2%)
Biofilm-haemagglutination-hypermucoviscosity*-*gelatinase-*BssS-fimH*	0 (0%)	1 (2%)
Biofilm-serum resistant-haemagglutination-hypermucoviscosity-*BssS-fimH*-*iss*	0 (0%)	2 (4%)
Biofilm-haemagglutination-hypermucoviscosity-lipase-*BssS-fimH*-*iss-iucA*	0 (0%)	1 (2%)
Biofilm-serum resistant-haemagglutination-hypermucoviscosity-lipase-*BssS-fimH*	0 (0%)	2 (4%)
Biofilm-serum resistant-haemagglutination-hypermucoviscosity-lipase-*BssS-fimH*-*iss*	0 (0%)	1 (2%)

*Total*	50 (100%)	50 (100%)
