# Corrigendum to “Association between Virulence Factors and Extended Spectrum Beta-Lactamase Producing* Klebsiella pneumoniae* Compared to Nonproducing Isolates”

**DOI:** 10.1155/2018/1023076

**Published:** 2018-01-08

**Authors:** Mustafa Muhammad Gharrah, Areej Mostafa El-Mahdy, Rasha Fathy Barwa

**Affiliations:** ^1^Microbiology & Immunology Department, Faculty of Pharmacy, Mansoura University, Mansoura 35516, Egypt; ^2^Department of Pharmaceutical Sciences, College of Pharmacy, Princess Norah Bint Abdulrahman University, Riyadh 11671, Saudi Arabia

In the article titled “Association between Virulence Factors and Extended Spectrum Beta-Lactamase Producing* Klebsiella pneumoniae* Compared to Nonproducing Isolates” [1], there were errors, which should be corrected as follows:

- In the Results section, “a total of twenty-four different virulence profiles were observed among the tested isolates,” should be “a total of twenty-three different virulence profiles were observed among the tested isolates.”
- In the Discussion section, “analysis of virulence factors combination has brought out 23 different virulence profiles including 2 to 8 virulence factors (Table 4),” should be “analysis of virulence factors combination has brought out 23 different virulence profiles including 2 to 9 virulence factors (Table 4).”
- In the eighteenth row of Table 4, “Biofilm-serum resistant-haemagglutination-lipase-*α*-hemolysis-BssS-fimH-iss” should be corrected to “Biofilm-serum resistant-haemagglutination-hypermucoviscosity-lipase-*α*-hemolysis-*BssS*-*fimH*-*iss*.” The correct table is shown as follows:

## Figures and Tables

**Table 4:** Virulence gene profiles associated with ESBLs and non-ESBLs producing *K. pneumoniae* isolates.

Virulence profiles	ESBLs producing isolates, number (%)	Non-ESBLs producing isolates, number (%)
Biofilm-haemagglutination-*BssS-fimH*	11 (22%)	12 (24%)
Biofilm-haemagglutination-hypermucoviscosity-*BssS-fimH*-*iss-iucA*	1 (2%)	1 (2%)
Biofilm-haemagglutination-*BssS-fimH*-*iss*	6 (12%)	1 (2%)
Biofilm-serum resistant-haemagglutination-*BssS-fimH*	7 (14%)	1 (2%)
Biofilm-serum resistant-haemagglutination-*BssS-fimH*-*iss*	14 (28%)	3 (6%)
Biofilm-*BssS-fimH*	1 (2%)	1 (2%)
Biofilm-serum resistant-haemagglutination-lipase-*BssS-fimH*	2 (4%)	0 (0%)
Biofilm-serum resistant-haemagglutination-*BssS-fimH*-*iss-iucA*	3 (6%)	0 (0%)
Biofilm-serum resistant-haemagglutination-lipase-*α* hemolysis*-BssS-fimH*	1 (2%)	0 (0%)
Biofilm-serum resistant-haemagglutination-*BssS-fimH*-*iucA*	1 (2%)	0 (0%)
*BssS-fimH*	1 (2%)	0 (0%)
Biofilm-serum resistant-haemagglutination-hypermucoviscosity*-BssS-fimH*-*iss-iucA*	1 (2%)	0 (0%)
Biofilm-haemagglutination-gelatinase-*BssS-fimH*	1 (2%)	1 (2%)
Hypermucoviscosity*-BssS-fimH*	0 (0%)	2 (4%)
Biofilm-haemagglutination-hypermucoviscosity-*BssS-fimH*	0 (0%)	18 (36%)
Biofilm-haemagglutination-hypermucoviscosity-*α* hemolysis-*BssS-fimH*	0 (0%)	1 (2%)
Biofilm-serum resistant-haemagglutination-hypermucoviscosity-*BssS-fimH*-*iss*-*iucA*	0 (0%)	1 (2%)
Biofilm-serum resistant-haemagglutination-hypermucoviscosity-lipase-*α*-hemolysis-*BssS*-*fimH*-*iss*	0 (0%)	1 (2%)
Biofilm-haemagglutination-hypermucoviscosity-gelatinase-*BssS-fimH*	0 (0%)	1 (2%)
Biofilm-serum resistant-haemagglutination-hypermucoviscosity-*BssS-fimH*-*iss*	0 (0%)	2 (4%)
Biofilm-haemagglutination-hypermucoviscosity-lipase-*BssS-fimH*-*iss-iucA*	0 (0%)	1 (2%)
Biofilm-serum resistant-haemagglutination-hypermucoviscosity-lipase-*BssS-fimH*	0 (0%)	2 (4%)
Biofilm-serum resistant-haemagglutination-hypermucoviscosity-lipase-*BssS-fimH*-*iss*	0 (0%)	1 (2%)

*Total*	50 (100%)	50 (100%)
